# Diagnostic value of implantable loop recorder in patients undergoing cryoballoon ablation of atrial fibrillation

**DOI:** 10.1111/anec.12733

**Published:** 2019-12-21

**Authors:** Aleksander Kusiak, Marek Jastrzębski, Adam Bednarski, Piotr Kułakowski, Roman Piotrowski, Edward Koźluk, Artur Baszko, Danuta Czarnecka

**Affiliations:** ^1^ 1st Department of Cardiology, Interventional Electrocardiology and Hypertension University Hospital in Krakow Krakow Poland; ^2^ College of Medicine 1st Department of Cardiology, Interventional Electrocardiology and Hypertension Jagiellonian University Krakow Poland; ^3^ Department of Cardiology Centre of Postgraduate Medical Education Grochowski Hospital Warsaw Poland; ^4^ Department of Cardiology Warsaw Medical University Warszawa Poland; ^5^ 2nd Department of Cardiology Medical University in Poznan Poznan Poland

**Keywords:** ablation, atrial fibrillation, cryoballoon ablation, ILR, implantable loop recorder

## Abstract

**Background:**

Due to limited data, implantable loop recorders (ILR) are not currently recommended by the guidelines to routinely monitor patients after atrial fibrillation (AF) ablation.

**Aims:**

To validate the diagnostic value of ILR after AF ablation, modern generation ILRs (LINQ) were implanted in patients scheduled for cryoballoon ablation of AF (CBA).

**Methods:**

We included 29 patients with frequent and symptomatic episodes of paroxysmal AF. ILR was implanted 3 months prior to CBA, and data were collected before and for 6 months after the procedure. The device was programmed to maximize sensitivity of AF/ atrial tachycardia (AT) detection. All EGM recordings were “manually” assessed and annotated as true AF, pseudo AF, unrecognized AF, and episodes with no EGM available. Duration and episode‐based standard performance metrics were evaluated.

**Results:**

A total number of 5,842 episodes were recorded. A total of 4,403 episodes were true AF, 453 episodes were pseudo AF, and 986 episodes had no EGM available. The device did not recognize 144 episodes of AF. Duration‐based sensitivity was 95.2%, duration‐based specificity 99.9%, duration‐based PPV 99.2%, duration‐based NPV 99.9%, episode‐based sensitivity 98.0%, and episode‐based PPV 91.0%. Misdiagnosis happened in 1 in 10 episodes. Total data review time was 166 hr.

**Conclusions:**

Implantable loop recorders is a valuable tool in evaluation of AF episodes in patients undergoing CBA. However, for high precision all recorded episodes need to be evaluated “manually.” The memory storage space is too low for frequent AF episodes, resulting in overwriting of stored EGMs and data loss.

**What's new?**

- For the first time, such a large number of arrhythmia episodes have been evaluated automatically by the device and verified by an observer.
- We showed that the data gathered by the device cannot be fully trusted as misclassifications were not infrequent.
- Some unique ILR recordings are presented.

## 1 INTRODUCTION

Implantable loop recorders (ILR) are currently recommended in various clinical situations when long‐term cardiac monitoring is needed. These devices have proven to be a useful tool in the diagnosis and treatment of heart palpitations, unexplained syncope, cryptogenic stroke, or ventricular arrhythmias. They also seem to be the perfect method of monitoring patients undergoing atrial fibrillation (AF) ablation (Pokushalov et al., 2011; Task Force Members, Brignole, Vardas, & Hoffman, 2^2009^). For obvious reasons, traditional methods of heart rhythm assessment as electrocardiogram (ECG) or 24‐hr Holter monitoring may not capture all AF episodes, with a considerable number of them being asymptomatic. It has been proven that the correlation between symptoms and occurrence of AF is poor (Verma et al., 2013). Although ILRs are not currently recommended by the guidelines to routinely monitor patients undergoing AF ablation, this looks like a promising method to precisely assess the AF recurrence rate after procedure. Accurate information regarding arrhythmia episodes, arrhythmia types, and correlation with symptoms could help indicate patients who need redo ablation and guide pharmacological treatment. To validate the clinical value of ILR after AF ablation, we decided to implant modern generation devices in patients scheduled for cryoballoon ablation of atrial fibrillation (CBA).

## 2 METHODS

The study enrolled 29 patients scheduled for CBA. ILR Reveal LINQ™ (Medtronic Inc.) was implanted 3 months prior to scheduled CBA. The device was inserted in an electrophysiology laboratory. It was placed in the left parasternal area at the level of the 4th−5th intercostal space 45 degrees to the sternal border, as instructed by the manufacturer. The incision and insertion tools supplied by the manufacturer were used. Patients received local anesthesia; no procedure‐related complications were observed (Wong et al., 2016). Patients were followed for the total time of 9 months after ILR insertion, consisting of 3 months prior and 6 months after CBA. The ILR was programmed to maximize sensitivity of AF/ atrial tachycardia (AT) detection to precisely asses the real number of AF episodes and calculate the burden of arrhythmia. ILRs were programmed as follows: AT/AF episodes = ALL, sensitivity = 35 µV and blanking = 150 ms, “ectopy rejection” algorithm = disabled. The device makes a rhythm classification every 2 min; therefore, it is able to detect AF lasting at least 2 min. Up to 14 episodes of AF can be stored with ECG tracing; afterward, the earliest episode is overwritten by most recent episodes. The longest AF episode is always preserved. The transmission of episodes from device memory was conducted daily via remote monitoring system. The device was able to record pauses with time duration longer than 3 s. In case of symptoms, patient could manually activate the ILR with a remote controller and store up to 4 episodes with time duration of 7.5 min (1 min prior and 6.5 min after device activation).

Cryoballoon ablation of pulmonary vein antra was performed under conscious sedation according to a standardized protocol. Briefly, after a single transseptal puncture with a Brockebrough needle each vein was occluded with the 28 mm cryoballoon (Arctic Front Advance, Medtronic Inc) under fluoroscopic visualization and with occlusion confirmed with the contrast method. Two 240‐s freezes were applied per vein, or more, if vein was not isolated with the first freeze; this was assessed with a circular catheter (Achieve, Medtronic Inc) using LAB System Pro electrophysiology system (Boston Scientific).

All patients were supplied with remote monitoring system (MyCareLink™, Medtronic Inc). Data stored by the device were daily sent to the Carelink^®^ web system. All data were evaluated on day‐to‐day basis by a clinician. ECG recordings of all AF episodes that were registered automatically and also ECGs of symptom‐related events were assessed and annotated as true AF or pseudo AF. All recordings were independently reviewed by two cardiologists, and in case of disagreement, third expert electrophysiologist was consulted.

### 2.1 Statistical analysis

All episodes lasting at least 2 min were analyzed and divided into true AF, false AF, unrecognized AF, and episodes with no EGM available. Duration and episode‐based standard performance metrics (sensitivity, specificity, positive predictive value [PPV], and negative predictive value [NPV]) were evaluated (Table 1.). Duration‐based metrics were calculated using time duration of true‐positive (TP), true‐negative (TN), false‐positive (FP), and false‐negative (FN) atrial fibrillation episodes. Episode‐based metrics calculated the proportion of true episodes and episodes unrecognized by the device. Episode PPV was calculated, but no NPV could be established, as a true‐negative episode cannot be defined (Sanders et al., 2016). Duration‐based metrics were also calculated for 3 months prior to and 6 months after CBA (Table 1.)

Episode sensitivity = Number of TPe/(Number of TPe + Number of FNe)
Episode PPV = Number of TPe/(Number of TPe + Number of FPe)
Duration sensitivity = TP Duration/(TP duration + FN Duration)
Duration specificity = TN Duration/(TN Duration + FP Duration)
Duration PPV = TP Duration/(TP Duration + FP Duration)
Duration NPV = TN Duration/(TN Duration + FN Duration)

**Table 1:** Duration and episode detection performance

Performance metrics	Overall	3 months prior to CBA	6 months after CBA
Duration‐based sensitivity (%)	95.2	94.9	96.4
Duration‐based specificity (%)	99.9	99.9	99.9
Duration‐based PPV (%)	99.2	99.3	97.8
Duration‐based NPV (%)	99.9	99.4	99.9
Episode‐based sensitivity (%)	98.0		
Episode‐based PPV (%)	91.0		

Abbreviations: CBA, cryoballoon ablation; NPV, negative predictive value; PPV, positive predictive value.

## 3 RESULTS

Twenty‐nine patients with paroxysmal atrial fibrillation resistant to antiarrhythmic drug therapy were included into the study (20 men, mean age 55.6 years old, range 34–72). All patients had uneventful ILR insertion and successful CBA (all 4 veins were isolated in every patient). CBA resulted in significant reduction of AF burden from 9.4% to 0.2%; the baseline characteristics of the enrolled patients and CBA outcomes are listed in Table 2.

**Table 2:** The characteristics of the enrolled patients (*n* = 29)

Patient characteristics	
Male (*n*)	20 (69%)
Age (years)	55.6
LV ejection fraction (%)	60.2
Left atrial size (mm)	36.8
Coronary heart disease (*n*)	4 (14%)
Hypertension (*n*)	15 (52%)
Atrial fibrillation burden pre CBA	9.4%
Atrial fibrillation burden post‐CBA	0.2%

Abbreviations: CBA, cryoballoon ablation; LV, left ventricle

All patients were followed for 3 months prior and 6 months after CBA. During the observation period, ILRs detected 5,842 episodes (8,920.3 hr) marked as AF. After “manual” assessment, 453 episodes (66.9 hr) (8%) were rejected as falsely diagnosed AF, and for 986 episodes (630.8 hr) (17%), there were no stored data (EGM) available. Therefore, the final number of AF true‐positive episodes that were possible to evaluate was 4,403 (8,222.6 hr). Additionally in 144 episodes (420.0 hr) initially diagnosed by the ILR as AT, the final diagnosis was changed to AF after evaluation.

**Figure 1:**
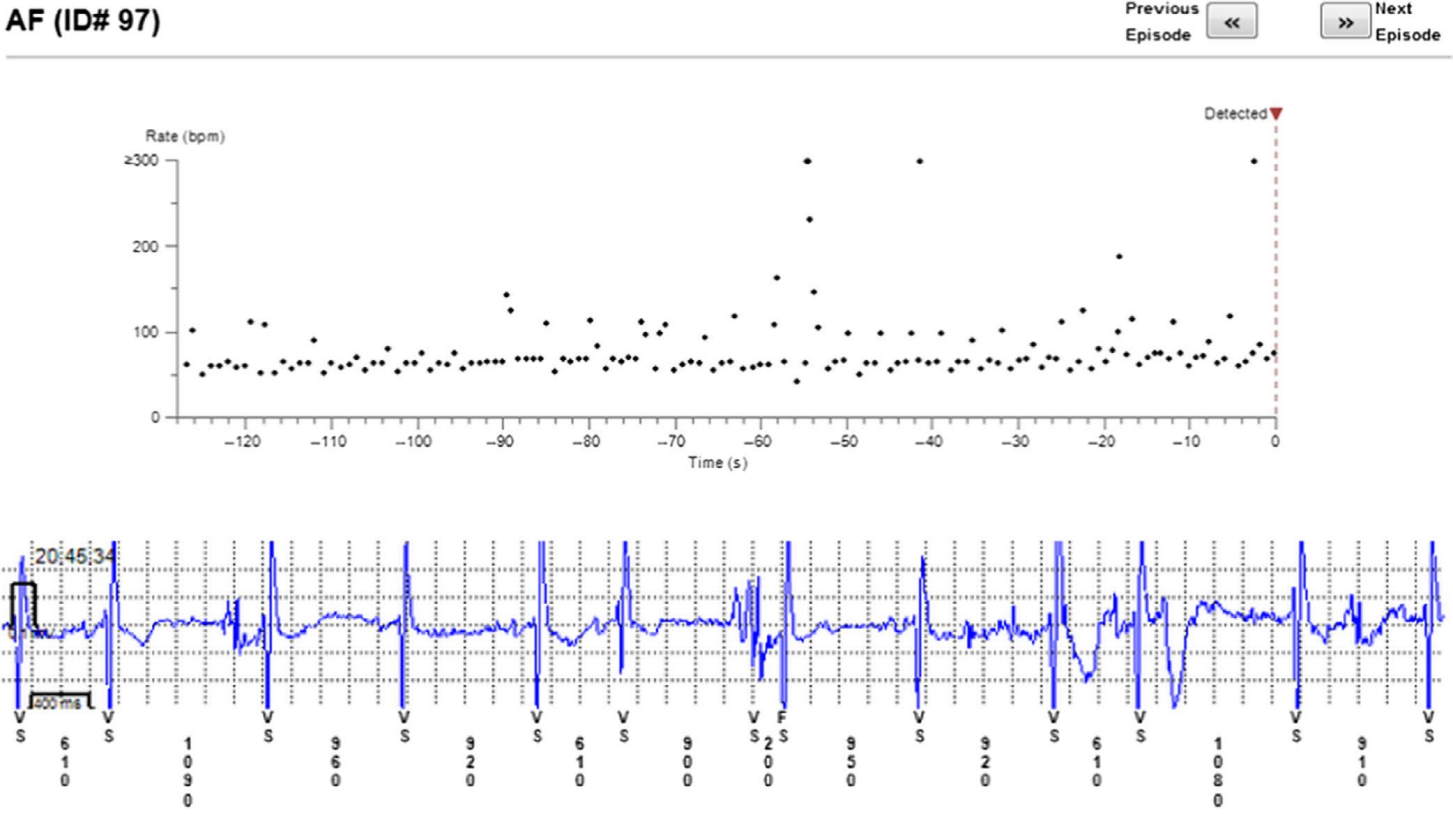
Incorrectly diagnosed AF. In fact sinus rhythm with extrasystoles and some noise

**Figure 2:**
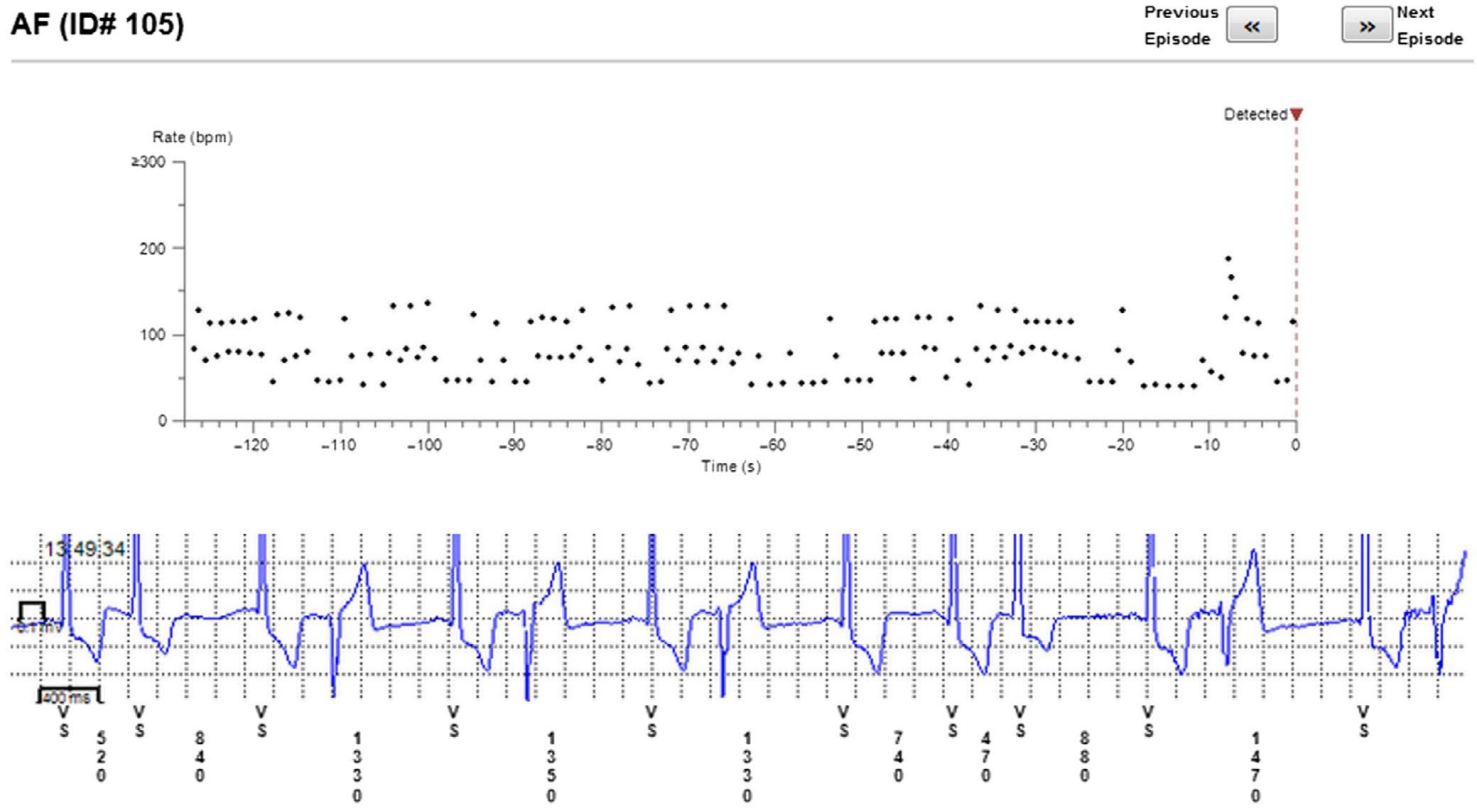
Incorrect diagnosis of AF due to extrasystoles

**Figure 3:**
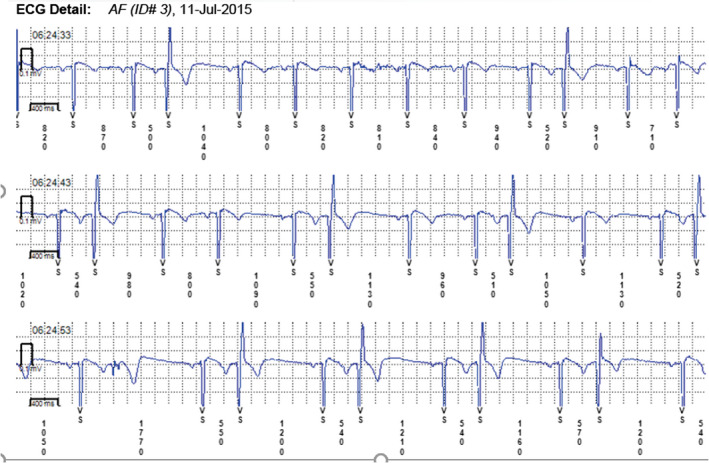
Extrasystoles diagnosed as AF

**Figure 4:**
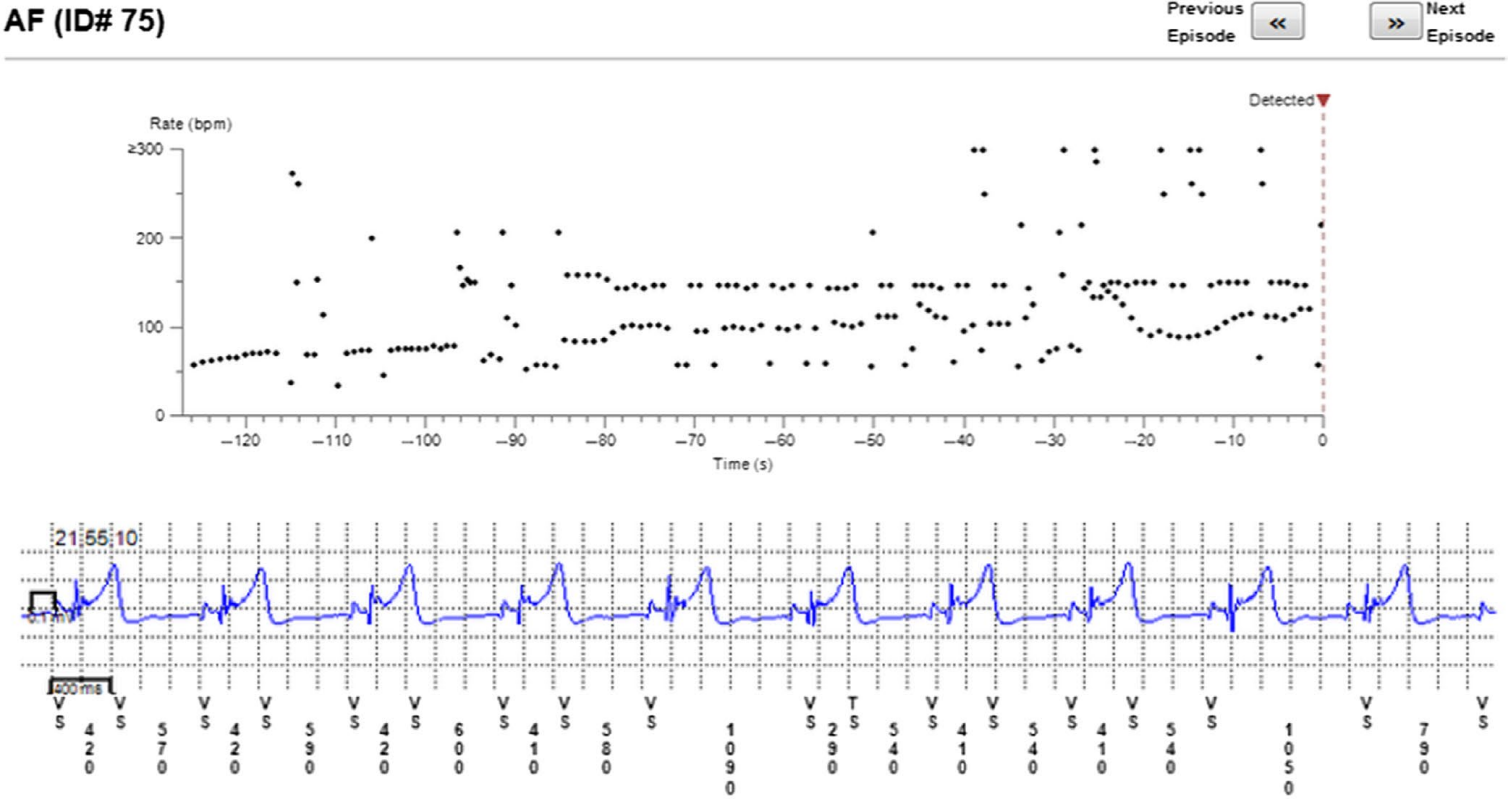
Incorrect diagnosis of AF due to T‐wave oversensing—railroad track pattern

The reason for incorrect diagnosis of AF was in most cases supraventricular extrasystoles or external electric interference (noise)—Figures 1, 2, and 3. There were also some episodes of T‐wave oversensing (Figure 4). The ILRs had mainly difficulties to differentiate arrhythmias in short episodes that lasted up to 4 min.

**Figure 5:**
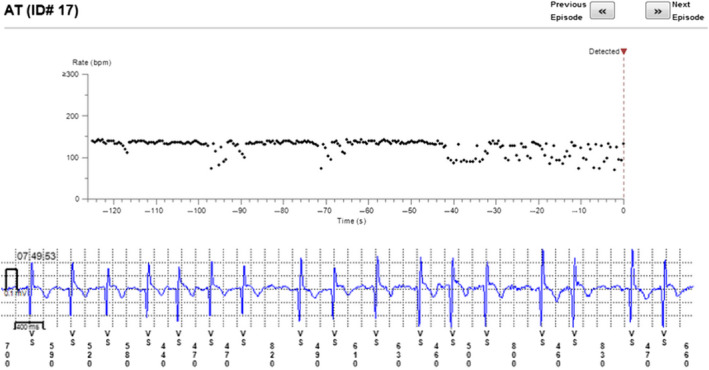
Episode of AF incorrectly diagnosed as AT. Two short episodes of AF (<2 min) present

**Figure 6:**
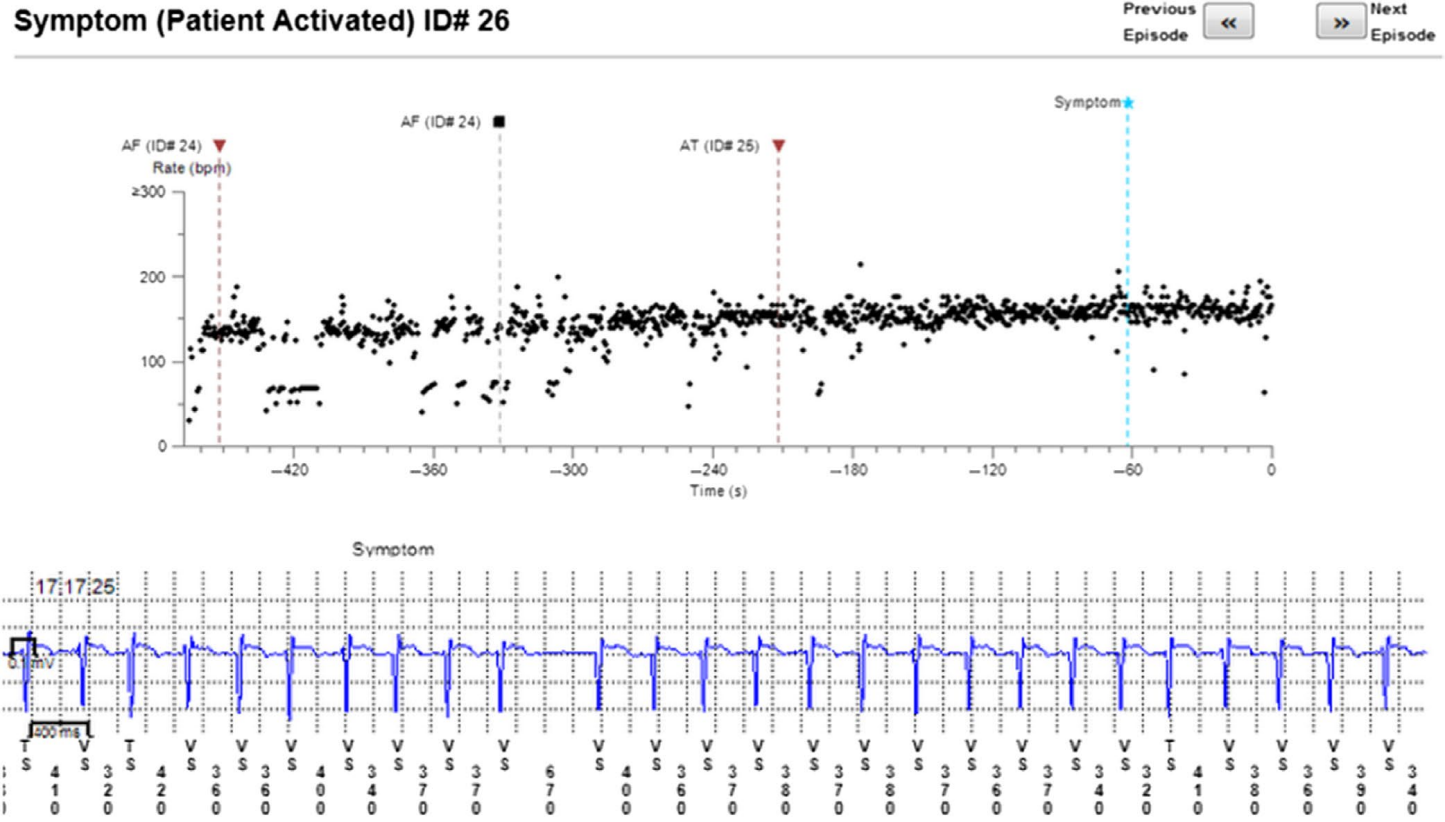
Patient‐activated recording shows AF and misdiagnosed AT (in fact AF)

**Figure 7:**
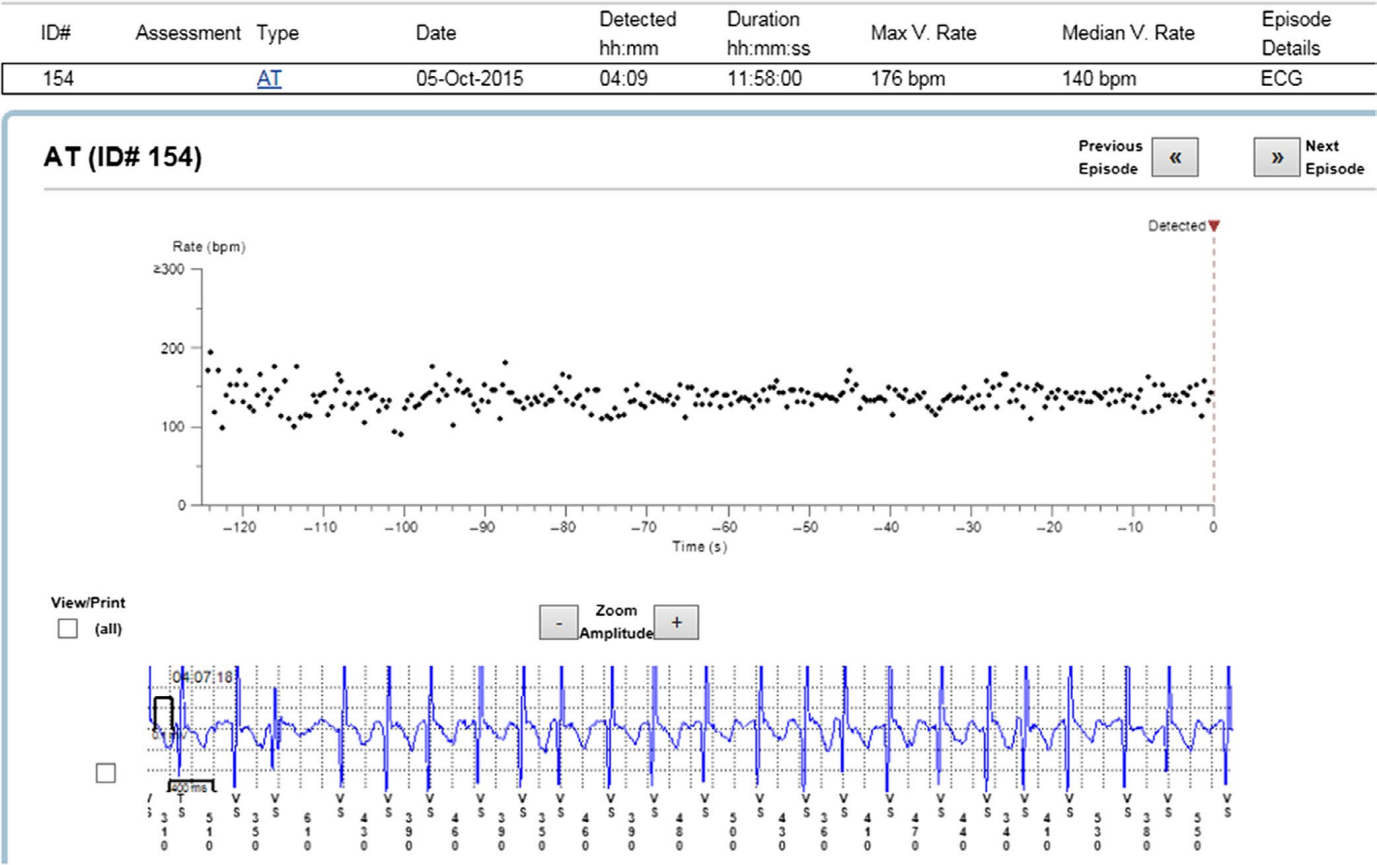
Episode of AF incorrectly diagnosed as AT

**Figure 8:**
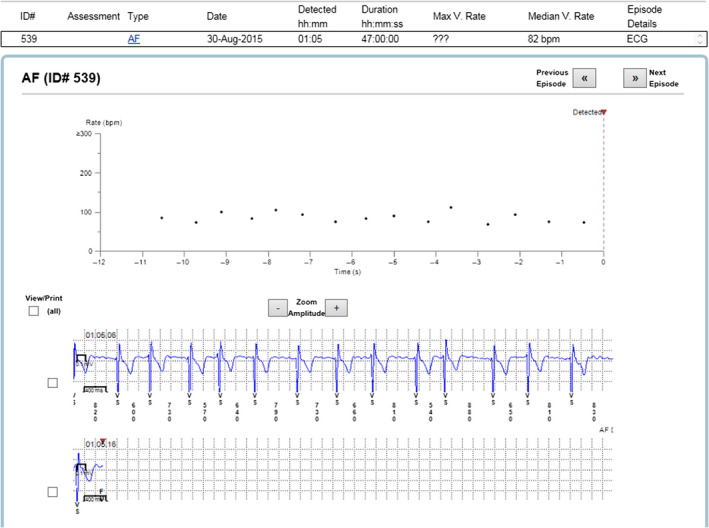
Example of episode lacking data

In some cases, the device could not recognize AF when it was present (Figures 5, 6, and 7). In total, the ILRs did not diagnose accurately 137 episodes of AF, diagnosing atrial tachycardia (AT) instead (Figure 8).

Patients were advised to send the data on daily basis, yet often the number of episodes recorded by the ILR was so high that the device was not able to store all EGMs. Therefore, it was not possible to “manually” assess all episodes.

It took approximately two minutes to adjudicate each available EGM. With total number of 5,000 EGMs, 10,000 min was required (about 166 hr) to evaluate them all.

The performance‐based metrics are summarized in Table 1.

## 4 DISCUSSION

In the current study, we assessed the clinical value of ILR in arrhythmia monitoring and categorization in patients undergoing CBA and showed that approximately 10% of recorded arrhythmia episodes were misclassified by the automatic, device‐based method. For precise and reliable arrhythmia assessment, all episodes needed to be verified “manually.”

In the current study, the most recent generation of ILR—Reveal LINQ was tested. Despite small size, it is packed with upgraded software when compared to older generations of ILR. The new generation device was supposed to overcome many issues reported for its predecessor (Gunda et al., 2015; Hindricks et al., 2010). It had very good duration‐based sensitivity and specificity (Table 1) that stands in line with earlier observations (Sanders et al., 2016). The longer the device was active and the longer were the AF episodes, the better were sensitivity and specificity. In our study, we observed patients with high number of AF episodes that transferred to large number of EGMs. This might be the reason for less false‐positive episodes than indicated in earlier studies (Damiano et al., 2016). To make the device more sensitive to AF, ectopy rejection algorithm was programmed off. In our study, we attempted to record each AF episode, even the short ones. Based on our observations and large trials’ data (e.g., CASTLE‐AF), in real‐life settings not every patient might need that precise assessment. We would suggest to routinely activate the ectopy ejection function. Possibly with this feature turned on, the number of wrongly diagnosed episodes would be lower. However, the ectopy was not the only reason the ILR was not able to correctly recognize AF episode. Based on our observations, there is no general ILR setting that would fit all patients; the programming of an ILR should be tailored to a particular clinical situation.

Personal assessment of multiple episodes daily in several patients is extremely time‐consuming. The assessment of all recorded episodes took 166 hr. In other words, time needed to evaluate all episodes in our study is equal to a week of constant work. Such a time commitment seems impossible in real‐life settings without dedicated team well trained in EGM interpretation. Based on our observations, it might be important issue preventing common usage of ILR in post‐CBA patients.

Moreover, limited storage capacity makes it impossible to evaluate all episodes. Even in patients conducting transmissions on daily basis, large number of episodes was overwritten by most recent ones. This problem could easily be solved by expanding the internal memory of the device. Another solution is to export the stored data in simplified format possible to be analyzed off‐line by other algorithms. With no recorded EGMs, there is no possibility to verify diagnosis made by ILR, and as we indicated, automatic diagnosis could not be fully trusted. One of possible solutions to this problem is to increase the device memory space. This would likely be beneficial for the patients, even if requires a moderate increase in the size of the device. Another storage expanding possibility is to collect and export raw data for further off‐line analysis. The ILR seems to have higher accuracy with longer episodes. Most problems regarding incorrect diagnosis were with episodes lasting 2 min. This observation is also in concordance with earlier studies (Sanders et al., 2016).

Current ESC guidelines define episode of AF as lasting at least 30 s (Lip et al., 2019). The 2‐min loop that is used to check heart rhythm by ILR will obviously skip some short episodes of arrhythmia. Those short runs of AF are not uncommon. They were often recorded when patient pressed symptoms button, and the device stored longer EGM. It is also not possible to evaluate how many clinically silent AF episodes lasting less than 2 min happened. Regarding issues with limited storage and inaccuracy when assessing short episodes, it is still frustrating that some AF episodes remain undetected despite continuous monitoring. The clinical significance of short AF episodes remains uncertain (Steinberg, O’Connell, Li, & Ziegler, 2018). The total number of AF episodes, including these lasting less than 2 min, is definitely greater than indicated by the ILRs.

### 4.1 Clinical implications

The traditional methods of postablation AF recurrence assessment like arrhythmia‐related symptoms, ECG, or 24‐hr ECG monitoring seem to be inadequate. It has already been proven that traditional follow‐up methods overestimate AF ablation success rate by up to 14% when compared to continuous monitoring with ILR (Gersak, Pernat, Robic, & Sinkovec, 2012).

Our observation stands in line with previous studies showing dramatic reduction of AF burden post‐CBA. It needs to be emphasized that the decision to ablate AF should be mainly based on the presence of arrhythmia symptoms; the ILR data are only complementary. However, ILR data can explain the nature of patient symptoms by providing a firm correlation; occasionally, AF‐like symptoms are caused by a different arrhythmia (mainly atrial extrasystoles in our experience), and AF ablation/re‐ablation would be a mistake. We believe that with 10% arrhythmia misclassification rate by the ILR, “manual” verification of automatic arrhythmia categorization should be considered when important decisions are made on the basis of ILR data. With reliable information regarding AF recurrences, it could be possible to decide in some patients with borderline CHA_2_DS_2_‐VASc scale score whether the anticoagulation therapy should be continued or might be stopped. It is still debatable whether a male with 1 point or a female with 2 points in that scale should be anticoagulated. In such cases, reliable ILR data could provide additional guidance. The same holds true for antiarrhythmic drugs use; accurate information about AF episodes, including asymptomatic ones, could guide optimal pharmacological treatment.

### 4.2 Limitations

The main limitation of the study is relatively small study group. However, in study population there was a large number of observed episodes.

## 5 CONCLUSIONS

ILR could be a valuable tool in AF burden assessment in patients undergoing CBA as it provides a detailed data about the arrhythmia occurrence. However, manual verification of episodes should be considered in some cases, as AF misdiagnosis was frequent—observed by us in almost 10% of recorded episodes.

## CONFLICT OF INTEREST

Authors report no conflict of interest regarding this article.

## AUTHORS' CONTRIBUTION

Conceived and designed the analysis: AK, MJ. Collected the data: AK, MJ, AB, PK, RP, EK, AB, DC. Contributed data or analysis tools: AK, AB. Performed the analysis: AK, AB, MJ. Wrote this paper: AK, MJ.

## ETHICS

All procedures performed in this study were in accordance with the ethical standards of the institutional and national research commitee and with the 1964 Helsinki declaration and its later amendments.
